# Supplementary material for: FOXN3 and GDNF Polymorphisms as Common Genetic Factors of Substance Use and Addictive Behaviors
Source: J Pers Med. 2022 Apr 26;12(5):690. doi: 10.3390/jpm12050690 (PMC9144496; doi:10.3390/jpm12050690)
Supplement: Supplementary file 1 [file jpm-12-00690-s001.zip › jpm-1687067-supplementary.pdf]

Table S1 Genotype distribution of the analyzed 32 SNPs

Gene	dbSNP number	Genotype	N	Observed frequency	Expected frequency[1]	HWE[2] p	Call rate
FOXN3	rs759364	AA	324	11%	9%		
		AG	1066	37%	41%	**<0.001**	96.94%
		GG	1521	52%	50%		
GDNF	rs3096140	AA	1327	51%	50%		
		AG	1062	40%	41%	0.589	87.35%
		GG	234	9%	9%		
GDNF	rs1549250	AA	965	33%	34%		
		AC	1456	50%	49%	0.314	96.90%
		CC	489	17%	17%		
GDNF	rs2910702	CC	162	6%	6%		
		CT	1061	36%	36%	0.977	97.30%
		TT	1699	58%	58%		
GDNF	rs11111	CC	66	2%	2%		
		CT	791	27%	27%	0.658	97.74%
		TT	2078	71%	71%		
GDNF	rs2973033	CC	225	8%	8%		
		CT	1188	41%	41%	0.854	96.64%
		TT	1489	51%	51%		
GDNF	rs3812047	CC	2105	76%	76%		
		CT	620	23%	22%	0.602	92.01%
		TT	38	1%	2%		
GDNF	rs1981844	CC	218	8%	8%		
		CG	1138	42%	41%	0.737	91.11%
		GG	1380	50%	51%		
CNR1	rs806380	AA	1327	45%	45%		
		AG	1296	44%	44%	0.827	98.43%
		GG	333	11%	11%		
CNR1	rs2023239	CC	59	2%	3%		
		CT	909	31%	29%	**<0.001**	97.44%
		TT	1958	67%	68%		
DRD1	rs4532	CC	402	14%	14%		
		CT	1423	48%	47%	0.346	98.17%
		TT	1123	38%	39%		
DRD2	rs6277	AA	823	28%	28%		
		AG	1481	50%	50%	0.932	98.30%
		GG	648	22%	22%		
ANKK1	rs1800497	AA	103	4%	4%		
		AG	933	31%	31%	0.749	98.70%
		GG	1928	65%	65%		

DRD3	rs6280	CC	286	10%	9%		
		CT	1233	42%	43%	0.821	97.30%
		TT	1403	48%	48%		
DRD4	rs1800955	CC	591	21%	21%		
		CT	1364	49%	50%	0.901	92.24%
		TT	815	30%	29%		
CHRNA5/A3	rs16969968	AA	351	12%	12%		
		AG	1304	46%	46%	0.996	95.14%
		GG	1202	42%	42%		
CHRNA5/A3	rs1051730	AA	403	14%	13%		
		AG	1235	43%	46%	**0.004**	95.70%
		GG	1236	43%	41%		
CHRNB3	rs6474412	CC	154	5%	5%		
		CT	1019	35%	35%	0.967	96.74%
		TT	1732	60%	60%		
OPRM1	rs1799971	AA	2277	77%	77%		
		AG	634	21%	21%	0.672	98.63%
		GG	51	2%	2%		
GABRA2	rs279858	CC	480	16%	16%		
		CT	1414	48%	48%	0.892	99.03%
		TT	1080	36%	36%		
TAS2R16	rs978739	CC	324	11%	12%		
		CT	1384	47%	45%	0.126	98.50%
		TT	1250	42%	43%		
FKBP5	rs1360780	CC	1487	52%	52%		
		CT	1115	39%	40%	0.322	94.64%
		TT	240	9%	8%		
FKBP5	rs4713916	AA	227	8%	7%		
		AG	1138	39%	40%	0.592	97.67%
		GG	1568	53%	53%		
ALDH2	rs886205	AA	1994	68%	68%		
		AG	864	29%	29%	0.891	98.10%
		GG	88	3%	3%		
ALDH1B1	rs2073478	GG	422	15%	15%		
		GT	1320	47%	47%	0.839	94.04%
		TT	1082	38%	38%		
ADH1C	rs698	CC	401	14%	15%		
		CT	1356	48%	47%	0.555	93.51%
		TT	1051	38%	38%		
ADH1C	rs1693482	CC	1087	37%	38%		
		CT	1396	48%	47%	0.727	96.70%
		TT	421	14%	15%		
FAAH	rs324420	AA	132	5%	5%	0.796	95.04%

		AC	992	35%	34%		
		CC	1730	60%	60%		
COMT	rs4680	AA	787	27%	28%		
		AG	1462	51%	50%	0.718	96.17%
		GG	639	22%	22%		
WFS1	rs1046322	AA	26	1%	1%		
		AG	558	19%	19%	0.559	98.57%
		GG	2376	80%	80%		
WFS1	rs9457	CC	980	33%	33%		
		CG	1420	48%	49%	0.585	98.43%
		GG	556	19%	18%		
CALD1	rs3800737	CC	290	10%	9%		
		CT	1241	42%	43%	0.741	98.24%
		TT	1419	48%	48%		

Notes. [1]Expected frequency based on the Hardy-Weinberg equilibrium; [2]Hardy-Weinberg equilibrium
